# Vitamin C Recycling Regulates Neurite Growth in Neurospheres Differentiated In Vitro

**DOI:** 10.3390/antiox9121276

**Published:** 2020-12-14

**Authors:** Francisca Espinoza, Rocío Magdalena, Natalia Saldivia, Nery Jara, Fernando Martínez, Luciano Ferrada, Katterine Salazar, Felipe Ávila, Francisco Nualart

**Affiliations:** 1 Laboratory of Neurobiology and Stem Cells, NeuroCellT, Department of Cellular Biology, Center for Advanced Microscopy, CMA BIO BIO, Faculty of Biological Sciences, University of Concepcion, Concepción 4030000, Chile; franespinoza@udec.cl (F.E.); rmagdalena@udec.cl (R.M.); nasaldivia@udec.cl (N.S.); neryalejara@udec.cl (N.J.); femartin@udec.cl (F.M.); luferrada@udec.cl (L.F.); katterinsalazar@udec.cl (K.S.); 2 School of Nutrition and Dietetics, Faculty of Health Sciences, University of Talca, Talca 3460000, Chile; favilac@utalca.cl; 3 Departamento de Biología Celular, Facultad de Ciencias Biológicas, Universidad de Concepción, Concepción 4070386, Chile

**Keywords:** vitamin C, retinoic acid, SVCT2, neuron, astrocytes, vitamin C recycling, GLUT1, bystander effect, neuronal differentiation, neurites

## Abstract

The reduced form of vitamin C, ascorbic acid (AA), has been related with gene expression and cell differentiation in the cerebral cortex. In neurons, AA is mainly oxidized to dehydroascorbic acid (DHA); however, DHA cannot accumulate intracellularly because it induces metabolic changes and cell death. In this context, it has been proposed that vitamin C recycling via neuron–astrocyte coupling maintains AA levels and prevents DHA parenchymal accumulation. To date, the role of this mechanism during the outgrowth of neurites is unknown. To stimulate neuronal differentiation, adhered neurospheres treated with AA and retinoic acid (RA) were used. Neuritic growth was analyzed by confocal microscopy, and the effect of vitamin C recycling (bystander effect) in vitro was studied using different cells. AA stimulates neuritic growth more efficiently than RA. However, AA is oxidized to DHA in long incubation periods, generating a loss in the formation of neurites. Surprisingly, neurite growth is maintained over time following co-incubation of neurospheres with cells that efficiently capture DHA. In this sense, astrocytes have high capacity to recycle DHA and stimulate the maintenance of neurites. We demonstrated that vitamin C recycling in vitro regulates the morphology of immature neurons during the differentiation and maturation processes.

## 1. Introduction

During the first weeks of postnatal development, cortical neurons undergo dynamic processes of neuronal maturation, such as dendritic spine morphogenesis and synapse formation [1,2,3]. On the other hand, astrocytes are formed from radial glial cells (RG) during the last days of embryonic development (E16–E21) and proliferate locally in the cerebral cortex between P2–P14 [4,5]. In postnatal weeks 3–4, astrocytes obtain a mature morphology [6], extending fine processes that will make contact with the synapses [7]. In recent years, attempts have been made to elucidate the mechanisms that relate astrocytic maturation and neuritic formation; however, the specific mechanisms by which astrocytes regulate neuronal morphology during early postnatal development are unknown.

The reduced form of vitamin C, ascorbic acid (AA), is transported at the cerebral level by the Sodium-dependent Vitamin C Transporter 2 (SVCT2); meanwhile, the oxidized form, dehydroascorbic acid (DHA), is transported by the glucose transporters 1 and 3 (GLUT1, GLUT3) [8,9,10]. SVCT2 expression analyses indicate that it is expressed in neurons [11,12,13], microglia [14] and Schwann cells [15]. Interestingly, SVCT2 is highly expressed during the first 5 postnatal days in the cerebral cortex, suggesting that neurons (in the process of arborization and maturation) require AA uptake. Additionally, SVCT2 overexpression increases N2a cell filopodia and lamellipodia, suggesting that AA may modulate these morphological changes [16,17]. Due to their high metabolic activity and consequent oxidative environment, cortical neurons rapidly oxidize AA to DHA inside the cell; however, DHA can induce important metabolic changes in cortical neurons, affecting two of the most important cellular energy pathways, glycolysis and the pentose pathway (PPP), while decreasing GSH levels [10,18]. Additionally, if cortical neurons are exposed to DHA under conditions of oxidative stress, cell death is induced, which is inhibited in the presence of astrocytes that can recycle DHA from the extracellular environment [19]. In this context, it has been postulated that neuron–astrocyte metabolic coupling allows for the maintenance of AA/DHA levels in the cerebral parenchyma, a process known as “vitamin C recycling”. In the vitamin C recycling mechanism, neurons capture AA through SVCT2 and oxidize it intracellularly to DHA, which is released to the extracellular medium through GLUT3. Subsequently, DHA is captured by astrocytes through GLUT1, so DHA is reduced to AA [20,21]. To date, this mechanism has only been described in the adult brain, in which neurons and astrocytes are fully mature; however, the role of vitamin C recycling during the early postnatal development of the cerebral cortex is unknown.

Nestin-positive cells present in neurospheres (NEs) grown in vitro under suspension conditions express the vitamin C transporter SVCT2 [12], and AA treatment elongates these cells, eventually resulting in the formation of embryonic brain radial glia cells [22]. However, in this work, we observed that adhered NEs exhibited neuronal differentiation and were responsive to vitamin C, increasing neurite formation. Remarkably, this phenotype was stronger with vitamin C treatment than with retinoic acid treatment, a classic inductor of neurogenic differentiation [23]. Thus, our in vitro experimental conditions seem to represent the conditions of the cerebral cortex during the first days of postnatal development [16]. Our work also demonstrates that vitamin C recycling, mediated by neuron–astrocyte interactions, regulates extracellular DHA concentrations such that DHA cannot inhibit neurite differentiation.

## 2. Materials and Methods

### 2.1. Animals

The animal care procedures were performed in accordance with the “Manual de Normas de Bioseguridad” (Comisión Nacional de Ciencia y Tecnología, CONICYT) and the Animal Care and Use Committee of the University of Concepcion (Chile). The experimental protocols were approved by the Concepción University Licensing Committee, grant number 1181243. The animals were housed under a 12-h light/dark cycle with food and water available ad libitum.

### 2.2. Primary and Cell Line Culture

Primary cultures of neurospheres were obtained from 17-day-old Sprague Dawley rat embryos. The cerebral cortex was dissected and mechanically disaggregated in neural stem cell (NSC) proliferation medium (Stem Cell Technologies, Vancouver, BC, Canada) supplemented with epidermal growth factor (EGF; 20 ng/mL), fibroblast growth factor (FGF; 10 ng/mL), and heparin (10 ng/mL) (Stem Cell Technologies). The cellular suspension was seeded in T25 cell culture flasks (BD Falcon^TM^, Flanklin Lakes, NJ, USA) at approximately 100,000 cells/cm^2^ to allow for neurosphere formation. After 2 days in vitro (DIV), the neurospheres were collected and adhered to poly L-lysine-coated dishes (0.1 mg/mL poly L-lysine; Sigma-Aldrich, St. Louis, MO, USA). Primary astrocyte cultures were obtained from the forebrains of 1- to 4-day-old postnatal Sprague-Dawley rats using standard protocols. HL60 cells were cultured in suspension at 1 × 10^6^ cells/mL in T75 flasks using Iscove’s modified Dulbecco’s medium (IMDM; Invitrogen, Waltham, MA, USA) with 10% FBS, penicillin 100 U/mL and streptomycin. The U87 cell line was maintained in DMEM supplemented with 10% FBS medium. For coculture assays, HL60 cells were added to 12-well plates with adhered NEs at a density of 100,000 cells/cm^2^. For U87 and cortical astrocyte cocultures, 25,000 cells/cm^2^ were adhered to 12-well poly L-lysine-coated dishes for 48 h before neurosphere adhesion

### 2.3. Cell Treatments, Viability and AA Concentration

Before all treatments, NEs were grown in suspension for 2 DIV and adhered to poly L-lysine-coated dishes. For the differentiation assays, L-AA (Sigma-Aldrich, St. Louis, MO, USA) was pre added to cultures at 0 (time of adhesion), 12, 24 and 48 h at a final concentration of 100 μM. PBS treatment was used as control. All-*trans*-retinoic acid (RA) (Sigma-Aldrich) dissolved in DMSO was added at the moment of adhesion at a final concentration of 10 μM. DMSO was used as control in these experiments. For DHA treatment, the NEs were treated with AA 100 μM at 0 and 12 h to induce differentiation. After that, dehydro-L-ascorbic acid dimer (Sigma-Aldrich, St. Louis, MO, USA) was added to cultures at 24 and 48 h at a final concentration of 100 μM. PBS was used as control. The culture medium was not changed during the treatments to allow DHA accumulation. Cell viability analysis was performed in NEs after 24, 48 and 72 h of treatment with AA 100 μM using an XTT cell proliferation kit (Biological Industries, Cromwell, CT, USA). AA concentration was measured by a FRASC colorimetric assay (#EASC-100, Bioassay system, Hayward, CA, USA), according to the manufacturer’s instructions.

### 2.4. Immunocytochemistry and Neurite Quantification

The cells were grown on glass cover slides in 24- or 12-well plates, fixed in 4% paraformaldehyde in phosphate-buffered saline (PBS) for 30 min, washed with Tris-HCl buffer (pH 7.8), and incubated in the same buffer containing 1% bovine serum albumin (BSA) and 0.2% Triton X-100 for 10 min at room temperature. Then, the samples were incubated with the following primary antibodies overnight at room temperature diluted in 1% BSA in Tris-HCl phosphate buffer: rabbit anti-GLUT1 (1:100, EMD Millipore, Burlington, MA, USA), mouse anti-βIII tubulin (Tuj1; 1:1000; Promega, Madison, Wisconsin, USA), rabbit anti-GFAP (1:400, Dako, Santa Clara, CA, USA), rabbit anti-Sox2 (1:100, Abcam, Cambridge, UK) and rabbit anti-SVCT2 (1:200, Novus Biologicals, Centennial, CO, USA). Next, the cells were incubated for 2 h with Cy2-, Cy3- or Cy5-labeled secondary antibodies at a 1:200 dilution (Jackson ImmunoResearch, West Grove, PA, USA), and nuclei were counterstained with Hoechst 33,342 (1:1000, Invitrogen, Waltham, MA, USA). The slides were analyzed using confocal laser microscopy (Confocal NLO 780, Carl Zeiss, Germany) and superresolution microscopy SIM-SR (ELYRA S1, Carl Zeiss, Germany) at CMA BIO-BIO (Concepcion University). For neurite volume quantification, images of processes positive for βIII tubulin at different planes were obtained from 6 NEs per condition using confocal microscopy. Imaris software (Bitplane, Belfast, Northern Ireland) was used to measure the volume of processes. In this analysis, the center of each neurosphere was excluded, and in each sample, four areas of 100 µm^2^ were selected at four locations (top, bottom, left and right) around the center. To calculate the percentage of NEs with neurites in one sample, 45–50 NEs were analyzed per condition; one neurite was considered a process positive for βIII tubulin longer than 10 µm (diameter of one cell into the NE).

### 2.5. Uptake Analysis

HL60 and U87 cells were used after 48 h of culture. Neurospheres were used after 24 h of adhesion, and astrocytes were cultured for 7 days. The cultures were selected carefully under a microscope to ensure that only plates with uniformly growing cells were used. The cells were washed and incubated with incubation buffer (15 mM HEPES, 135 mM NaCl, 5 mM KCl, 1.8 mM CaCl_2_, and 0.8 mM MgCl_2_) for 10 min at 37 °C. Uptake assays were performed in 500 μL of incubation buffer containing a final concentration of 100 μM 1-^14^C-L-AA (specific activity of 2,6 mCi/mmol; PerkinElmer, Waltham, Massachusetts, USA) and 0.1 mM DTT. Before the DHA uptake assays, 1-^14^C L-AA was oxidized to DHA by adding 0.02 U ascorbic acid oxidase/mL for 5 min at 37 °C. The uptake was stopped by washing the cells twice with ice-cold incubation buffer consisting of 0.8 mM HgCl_2_. The cells were lysed in 0.5 mL of lysis buffer (10 mM Tris-HCl [pH 8.0] and 0.2% SDS), and the incorporated radioactivity was assayed by liquid scintillation spectrometry. In some experiments, the cells were treated with 20 µM cytochalasin B (Sigma-Aldrich, St. Louis, MO, USA) or 5 µM WZB117 (Sigma-Aldrich, St. Louis, MO, USA) as described in the figure legends.

### 2.6. Reverse Transcription Polymerase Chain Reaction

Total RNA was isolated from neurospheres, HL60 cells, U87 cells, and astrocytes using TRIzol (Invitrogen, Waltham, MA, USA). For reverse transcription polymerase chain reaction (RT-PCR), 2 μg of RNA was included in a 20-μL reaction volume containing 5xM-MuLV reverse transcriptase buffer, 20 U of RNAse inhibitor, 1 mM dNTPs, 2.5 mM oligo(dt)18 primer, and 10 U of RevertAidTM H minus M-MuLV reverse transcriptase (Fermentas International Inc., Burlington, Ontario, Canada). The reaction mixture was incubated for 5 min at 37 °C, followed by 60 min at 42 °C and 10 min at 70 °C. For amplification, 1 μL of complementary DNA (cDNA) was included in a 12.5-μL reaction containing 10X PCR buffer without MgCl_2_ and using standard protocols. The following sets of primers were used: rGLUT1, sense 5′-TCAAACATGGAACCACCGCT-3′ and antisense 5′-AGAAACCCATAAGCACGGCA-3′ (expected product, 203 bp); hGLUT1 sense 5′-GTGGAGACTAAGCCCTGTCG-3′ and antisense 5′-GATGGGAAGGGGCAAATCCT-3′ (expected product, 200 bp); and mrSVCT2, sense 5′-TGCCAGGAAGGGTGTACTTC-3′ and antisense 5′-CCGGTACCAAATATGCCATC-3′ (expected product, 255 bp).

### 2.7. Western Blot Analysis

Total protein extracts were obtained from neurospheres and astrocytes, as well as from HL60 and U87 cells. To prepare total protein extracts, the cells were trypsinized and centrifuged, and the resultant pellet was homogenized in NP40 cell lysis buffer (Invitrogen^TM^, Waltham, MA, USA) with a protease/phosphatase inhibitor cocktail (1X, Cell Signaling Technology, Danvers, MA, USA) for 30 min at 4 °C and separated by centrifugation at 10,000 rpm for 10 min at 4 °C. Proteins were resolved by SDS-PAGE (40–75 μg/lane) in a 10% (*w*/*v*) polyacrylamide gel and transferred to PVDF membranes (0.45 µm, Immobilon-P, Merck Millipore, Germany); membranes were blocked and incubated overnight at 4 °C with rabbit anti-GLUT1 (1:5000, EMD Millipore, USA) or rabbit anti-SVCT2 (1:1.500, Novus Biologicals, Centennial, CO, USA) antibodies. Immunoreactive proteins were detected using enhanced chemiluminescence reagents according to the manufacturer’s instructions (Western Lighting^®^ Plus-ECL, Perkin Elmer, Waltham, MA, USA).

### 2.8. Immunodetection of Carboxymethyllysine (CML) and Carbonylated Proteins

The measurement of CML and carbonylated protein levels among intracellular proteins was performed by immunoblotting using an anti-CML primary antibody (R&D systems, Minneapolis, MN, USA) and the OxyBlot^TM^ Protein Oxidation Detection Kit (Chemicon International, Temecula, CA, USA), respectively. Cell lysates were obtained from cell cultures using CelLytic^TM^ (Sigma-Aldrich), and proteins were quantified using the BCA method using BSA as the standard protein [24]. Proteins (15 µg) were derivatized to 2,4-dinitrophenylhydrazone (DNP-hydrazone) by incubating with 2,4-dinitrophenylhydrazine (DNPH). For SDS-PAGE or CML analyses, proteins (22.5 µg) were boiled (5 min) in 62.5 mM Tris buffer, pH 6.8, containing 2% SDS, 10% glycerol and 100 mM β-mercaptoethanol as a reducing agent with traces of bromophenol blue as a tracking dye. SDS-PAGE samples were stained with a Coomassie brilliant blue solution R-250 (BIORAD, USA). For the CML and DNP analyses, the proteins were transferred to a nitrocellulose membrane (Pierce, Rockford, IL, USA). The membranes were incubated with 1% BSA and PBS-Tween 20 (0.05%) overnight. CML samples were incubated with an anti-CML primary antibody (1:1000) in PBS-Tween 0.05% with 1% BSA (blocking solution) for 1 h. DNP proteins were incubated with a rabbit polyclonal antibody (1:5000) specific for the DNP moiety of the proteins for 1 h in blocking solution at room temperature. After several washes in 0.05% PBS-Tween 20, the membranes were incubated with a goat horseradish peroxidase-conjugated anti-rabbit IgG (1:10,000) directed against the primary antibody in 0.05% PBS-Tween 20 with 1% BSA for 1 h at room temperature. After incubation with a chemiluminescent reagent (Western Lightning Chemiluminescence Reagent, Perkin Elmer-Life Sciences, Boston, MA, USA), the membranes were exposed to ECL films (Hyperfilm ECL, Amersham Biosciences, Les Ulis, France).

### 2.9. Flow Cytometry

Samples were obtained from neurospheres at different times of treatment. Cells were dissociated using trypsin/EDTA for 5 min at 37 °C and centrifuged for 10 min at 1200 rpm. The cells were resuspended in DMEM/F12 10% *v*/*v* FBS (Gibco, Waltham, MA, USA) and incubated with the following dyes: intracellular GSH (1X, Immunochemistry Technologies, Bloomington, MN, USA), CellROX^®^ (1 µM, Invitrogen, Waltham, MA, USA), SYTOX™ AADvanced™ (1 µM, Invitrogen, Waltham, MA, USA) or SYTOX^®^ Green (1X, Invitrogen, Waltham, MA, USA). After 15 min of incubation, the cells were analyzed using BD FACSAria III (BD Biosciences, Piscataway, NJ, USA), and positive cells were counted in each cell suspension by flow cytometry analysis in the phycoerythrin (PE) channel and fluorescein isothiocyanate (FITC) channel, depending on the dye. Data analysis was performed using FlowJo (Tree Star).

### 2.10. Statistical Analysis

The data represent the mean ± SEM with *n* = three independent experiments obtained from three independent biological samples. Statistical analyses were performed using GraphPad Prism version 6.01. Statistical comparisons between two groups were performed using parametric Student’s *t*-tests, and for more than two groups, analyses were carried out using parametric analysis of variance (ANOVA) followed by a Tukey posttest. *p* ≤ 0.05 was considered significant.

## 3. Results

### 3.1. NEs Adhered In Vitro Were Mainly Formed by Immature Neurons Positive for βIII Tubulin and SVCT2

**Figure 1:**
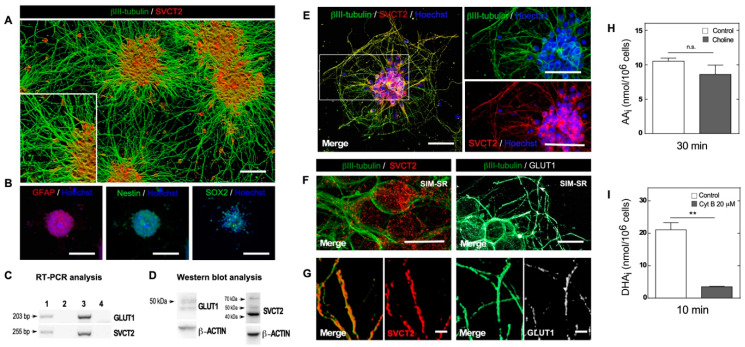
Adhered neurospheres showed SVCT2 expression mainly in differentiated neuronal cells. (**A**) Confocal microscopy with tile-scan 3D and rendering analysis of NEs stained with anti-SVCT2 (red) and anti-βIII tubulin (green). Inset. A detailed analysis of neurosphere processes. Scale bar, 100 µm. (**B**) NE confocal microscopy analysis using antibodies against GFAP, nestin, and SOX2 and nuclear staining with Hoechst. Scale bar, 50 µm. (**C**) PCR analysis of SVCT2 and GLUT1 mRNA. Line 1: cDNA obtained from control NEs adhered for 24 h. Line 2: Control reaction without reverse transcriptase. Line 3: Postnatal day 8 rat brain cDNA (positive control). Line 4: Negative control (water). (**D**) Western blot analysis with anti-GLUT1 and anti-SVCT2 antibodies in extracts of control NEs 24 h postadhesion. (**E**) Confocal microscopy and merge analysis using anti-SVCT2 (red) and anti-βIII-tubulin (green) antibodies and nuclear staining with Hoechst. Right side. Digital zoom images of the depicted zone in E. Scale bar, 50 µm. (**F**) SVCT2 and GLUT1 transporter detection by 3D-SIM superresolution (SIM-SR) microscopy analysis in control NEs adhered for 24 h. Left side. βIII-tubulin (green) and SVCT2 (red) detection. Right side. βIII-tubulin (green) and GLUT1 (white) detection. Scale bar, 10 µm. (**G**) SVCT2 and GLUT1 transporter detection by SIM superresolution (SIM-SR) microscopy analysis in control NEs adhered for 24 h. Left side. Digital magnification of βIII tubulin/SVCT2 positive processes. Right side. Digital magnification of βIII tubulin/GLUT1 positive processes. Scale bar, 2 µm. (**H**) AA uptake in NEs adhered (24 h, control) in the presence or absence of sodium. All data are representative of three separate experiments. Parametric Student’s *t*-test, n.s., not significant. (**I**) DHA uptake analysis in NEs adhered (24 h, control) in the absence or presence of 20 µM cytochalasin B (GLUT1 inhibitor). All data are representative of three separate experiments. Parametric Student’s *t*-test, ** *p* < 0.01.

We used NEs obtained from the cerebral cortex of a 17-day-old embryonic rat (E17), from which NSCs were obtained. In this work, we attached the NEs to a coated poly L-lysine dishes, and generated neuritic outgrowths positive for βIII tubulin (Figure 1A and inset). These processes were longer than the diameter of the cells present at the NE but were not clearly axons or dendrites; therefore, we referred to them as neurites. Inside the NE, the positive cell population was detected with anti-GFAP, and a large number of undifferentiated cells were positive for nestin and SOX2 (Figure 1B). Subsequently, we analyzed the expression and function of transporters for both the reduced and oxidized form of vitamin C, AA and DHA, respectively. Thus, we confirmed SVCT2 and GLUT1 expression by using RT-PCR and Western blot analysis (Figure 1C,D). An intense band of 42 kDa representing SVCT2 and two bands with a lower intensity of 50 and 70 kDa were observed (Figure 1D). Interestingly, when analyzing SVCT2 transporter localization, we observed that after 24 h of adhesion, cells with a neuronal phenotype were positive for βIII tubulin and expressed the SVCT2 transporter (Figure 1E). Using superresolution SIM microscopy (SIM-SR) analysis, we observed that SVCT2 was less represented in the cell membrane of neuronal somas (Figure 1F); however, SVCT2 generated a more regular positive reaction at the cellular processes (Figure 1G, SVCT2). GLUT1 had a homogeneous distribution in the cell membrane of immature neurons (Figure 1F), and was intensely detected in neurites (Figure 1G, GLUT1).

Finally, AA uptake assays showed an effective uptake of over 30 min, which was surprisingly not inhibited by the absence of sodium (Figure 1H), while there was a significant uptake of DHA over 10 min, which was inhibited by the presence of cytochalasin B (Figure 1I). Thus, adhered NEs expressed both functional AA and DHA transporters; consequently, they represented a postnatal development stage of different neurons present in the cerebral cortex [16].

### 3.2. Prolonged AA Neurosphere Treatment Induced the Loss of Cellular Neurites

**Figure 2:**
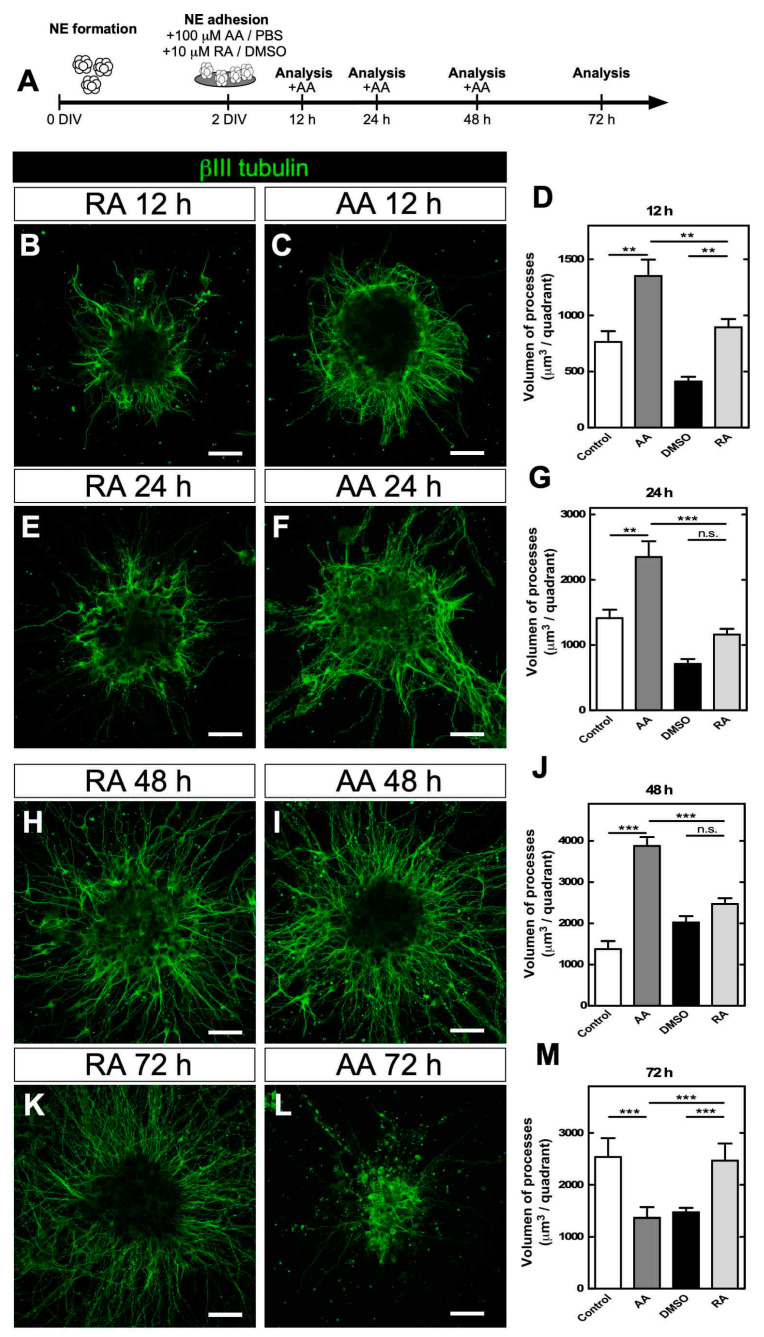
AA stimulated neuronal differentiation in adhered NEs. (**A**) Protocol for neuronal differentiation stimulated by AA or RA in adhered NEs. (**B**,**E**,**H**,**K**) Immunocytochemistry analysis of βIII tubulin (green) in NEs treated with 10 µM RA. Scale bar, 30 µm. (**C**,**F**,**I**,**L**) Immunocytochemistry analysis of βIII tubulin (green) in NEs treated with 100 µM AA for 24 to 72 h. Scale bar, 30 µm. (**D**,**G**,**J**,**M**) Neurite volume quantification. All data are representative of three separate experiments. Parametric ANOVA statistics analysis, Tukey posttest, ** *p* < 0.01; *** *p* < 0.001; n.s., not significant.

**Figure 3:**
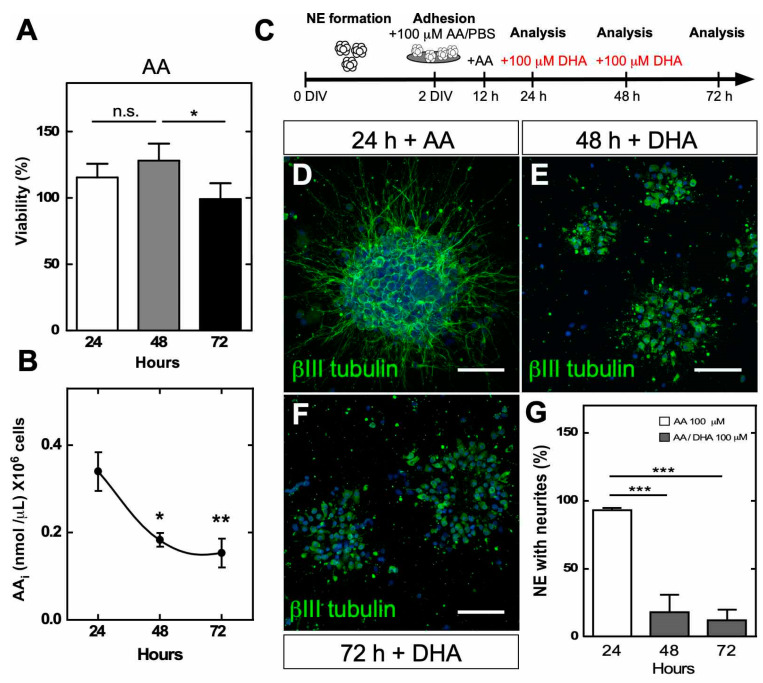
Treatment of NEs differentiated in vitro with DHA impacted neuritic growth. (**A**) Viability analysis of NEs treated with AA for 24, 48 and 72 h. All data are representative of three separate experiments. Nonparametric ANOVA statistical analysis, Tukey posttest, * *p* < 0.05; n.s., not significant. (**B**) Intracellular AA concentration during AA treatment. All data are representative of three separate experiments. Parametric ANOVA statistical analysis, Tukey posttest, * *p* < 0.05; ** *p* < 0.01. (**C**) DHA treatment protocol used in NE adhesion. (**D**–**F**) βIII tubulin (green) analysis in NEs treated with 100 µM AA for 24 h (**B**) or 100 µM AA for 24 h followed by 100 µM DHA until 48 h (**E**) or until 72 h (**F**). Scale bar, 60 µm. (**G**) NE neurite quantification at each treatment time point. A “neurite” was considered a cellular extension positive for βIII tubulin and at least 10 µm in length. All data are representative of three separate experiments. Parametric ANOVA statistical analysis, Tukey posttest, *** *p* < 0.001.

Considering that AA can induce neuronal differentiation in precursor cells [12,25] and that there is a high AA concentration in the cerebral cortex [21], we evaluated the effects of AA during the induction of neuronal differentiation. Adhered NEs were supplemented with 100 µM AA for 72 h, and the effect of AA was compared with that of RA, a molecule used as a positive control (Figure 2A). Through immunocytochemistry, we observed significant neuritic growth (βIII tubulin-positive cells) from 12 h posttreatment. Remarkably, neuritic growth in neurons was stronger with AA than that with RA (Figure 2B–D), a result that was maintained at 24 and 48 h posttreatment (Figure 2E–J). However, extended treatment with AA for 72 h drastically changed neurite morphology with fewer βIII tubulin-positive processes (Figure 2K,L) and significantly lower process volume than observed under the control condition (Figure 2M). The viability of the cultures was analyzed, and a decrease was not detected between 24 and 72 h (Figure 3A). Therefore, the observed morphological change was not associated with cell death. When measuring intracellular levels of AA, we observed that compared to 24 h of treatment, 48 and 72 h of treatment significantly decreased AA levels (reduced form) (Figure 3B). These results are suggestive of high intracellular oxidation of AA towards DHA, a form of vitamin C that would accumulate intracellularly.

To check whether DHA induced a decrease in the number of neurites, AA treatment was performed during the first 12 h, and then the NEs were directly supplemented with 100 µM DHA (Figure 3C). Morphological analysis showed that the NEs maintained their neurites up to 24 h (Figure 3D); however, from 48 h onwards, NEs without neurites were detected, a condition that increased up to 72 h (Figure 3E,F). Quantification of total NEs with neurites showed that almost 100% of NEs had neurites up to 24 h, which decreased by 80% after DHA treatment by 48 and 72 h (Figure 3G). These results indicated that direct treatment with DHA reproduced the phenotype observed with prolonged treatment with AA, suggesting that the oxidized form of vitamin C induced this change in neuronal morphology.

### 3.3. Vitamin C Recycling In Vitro Recovers Neuritic Morphology after Prolonged Treatment with AA

**Figure 4:**
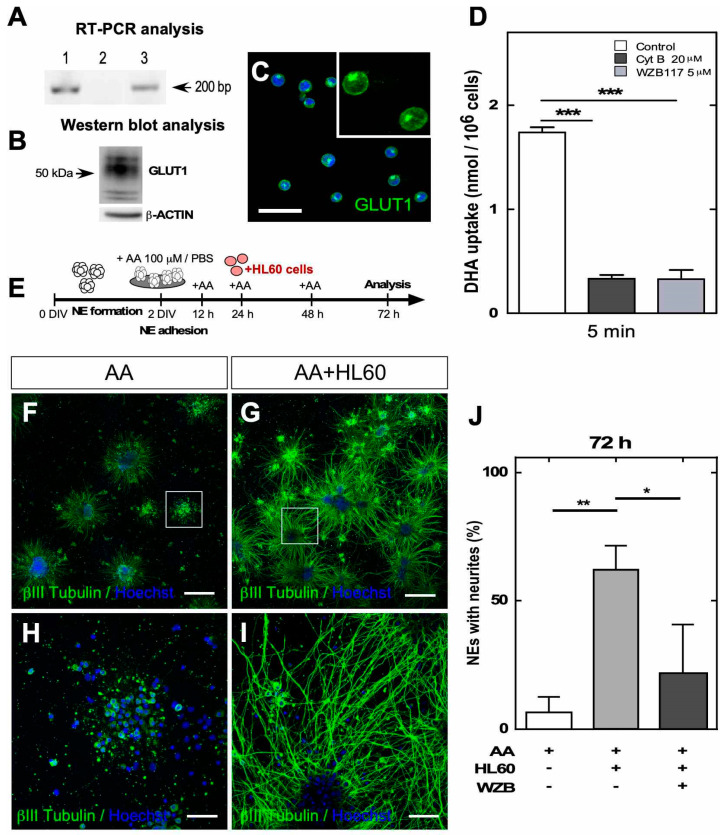
Coculture of HL60 cells with NEs maintained neurites in vitro after AA treatment. (**A**) PCR analysis of GLUT1 mRNA. RNA extracts obtained from HL60 cells (line 1), negative RT-PCR control (line 2), and positive control using human U87 cell line samples (line 3). (**B**) Western blot analysis for GLUT1 expression in HL60 cells. (**C**) GLUT1 immunocytochemical detection in HL60 cells. Scale bar, 30 µm. (**D**) Uptake of 100 µM DHA for 5 min in the presence of Na^+^, 20 µM cytochalasin B or 5 µM WZB117 (GLUT1 specific inhibitor). All data are representative of three separate experiments. Statistical analysis, parametric ANOVA test, followed by Tukey’s test, *** *p* < 0.001. (**E**) Protocol used to recycle vitamin C with HL60 cells. (**F**,**G**) Immunocytochemistry analysis of βIII tubulin in neurospheres treated with AA for 72 h without (**F**) or cocultured with HL60 cells after 24 h of treatment (**G**). Scale bar, 100 µm. (**H**,**I**) High-power image of NEs depicted in (**F**,**G**), respectively. Scale bar, 50 µm. (**J**) Quantification of the neurites of NEs until 72 h of treatment with 100 µM AA in the presence of HL60 cells or the WZB117 inhibitor (WZB). All data are representative of three separate experiments. Statistical analysis, parametric ANOVA test, followed by Tukey’s test, * *p* < 0.05; ** *p* < 0.01.

To verify that vitamin C recycling is important during neurite generation in vitro, we first used GLUT1-positive HL60 cells (Figure 4A,B), in which the GLUT1 transporter is mainly located in the cellular membrane (Figure 4C). We also observed that HL60 cells incorporated DHA and that the uptake was inhibited by cytochalasin B and WZB117, a specific inhibitor of GLUT1 [26] (Figure 4D). Subsequently, NEs were treated with AA for 24 h to allow DHA generation and release into the extracellular medium (Figure 4E). Then, we cocultured NEs with HL60 cells for 48 h (total time, 72 h), and neurite formation was analyzed. The HL60/NE cocultures showed increased βIII tubulin-positive processes (Figure 4G–I) compared with the control condition (NEs without HL60 cells) (Figure 4F–H). Quantitatively, only 10% of the NEs presented neurites when they were treated with AA and analyzed at 72 h. However, approximately 60% of the NEs in the NE/HL60 cocultures had neurites, which decreased significantly (25%) when HL60 cells were treated with a GLUT1 inhibitor (Figure 4J).

**Figure 5:**
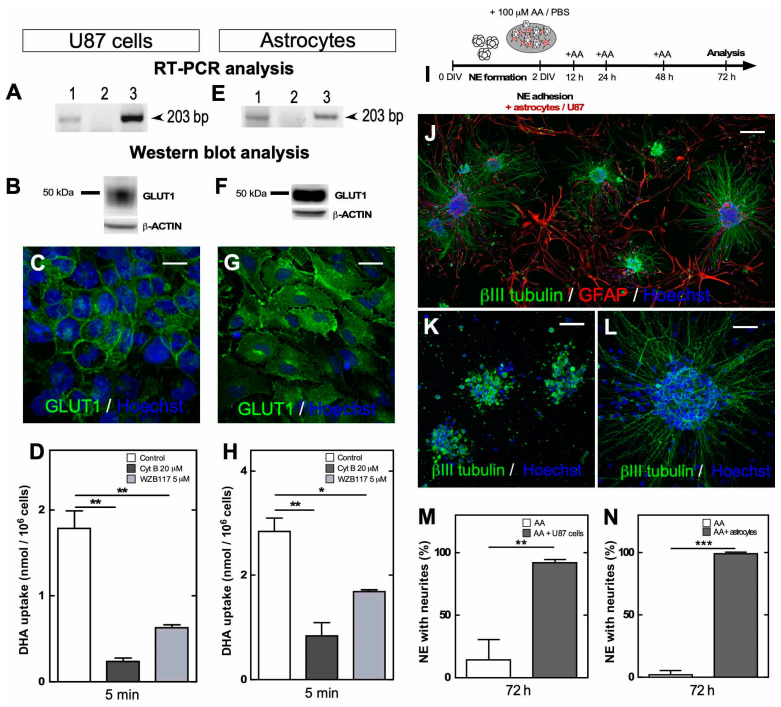
Vitamin C recycling using astrocytes, maintained neurite outgrowth under long-term AA treatment. (**A**,**E**) PCR analysis of GLUT1 mRNA. RNA extracts obtained from U87 cells (**A**) and cortical astrocytes (E)(line 1), negative RT-PCR control (line 2), and positive control using complete brain extract (line 3). (**B**,**F**) Western blot analysis of GLUT1 in U87 cell (**B**) and cortical astrocyte extracts (**F**). (**C**,**G**) Immunocytochemistry analysis of GLUT1 (green) in U87 cells (**C**) and astrocytes (**G**). Scale bar, 15 µm. (**D**,**H**) Uptake of 100 µM DHA for 5 min in the presence of 20 µM Na^+^, cytochalasin B or WZB117 in U87 cells (**D**) and astrocytes (**H**). All data are representative of three separate experiments. Parametric ANOVA, followed by Tukey’s test, * *p* <0.05; ** *p* <0.01. (**I**) Experimental model of mixed cultures of NEs with U87 cells or cortical astrocytes at 7 DIV, which were treated with AA for 72 h. (**J**,**L**) Immunocytochemical and confocal tile-scan microscopy analyses of βIII tubulin (green), GFAP (red) and nuclear staining with Hoechst, in mixed NEs cultured with cortical astrocytes. Scale bar, 100 µm. High-power image of NEs (**L**). Scale bar, 30 µm. (**K**) Immunocytochemical and confocal microscopy analyses of βIII tubulin (green) and nuclear staining with Hoechst, in control NEs treated with AA for 72 h in the absence of astrocytes. Scale bar, 30 µm. (**M**,**N**) Total NEs with neurites generated at 72 h in mixed cultures of NEs with U87 cells (**M**) or cortical astrocytes (**N**). All data are representative of three separate experiments. Parametric Student’s *t*-test was used for statistical analysis, ** *p* < 0.01; *** *p* < 0.001.

The same type of experiment was performed with U87 cells (glioblastoma cells) and astrocytes isolated from the brain cortex to define whether glial cells can generate a “bystander effect” [8] and participate in vitamin C recycling (Figure 5). Both cell types express GLUT1 (Figure 5A,B,E,F), and confocal microscopy showed that GLUT1 is mainly located in the cell membrane (Figure 5C,G). Then, we showed that the cells uptake DHA and that transport was inhibited with cytochalasin B or WZB117 (Figure 5D,H). As U87 cells or astrocytes adhered to the culture plate, NEs were seeded on astrocytes or U87 cells for recycling experiments and then treated with AA (Figure 5I,J). When performing immunocytochemical analyses at 72 h posttreatment, we observed that U87 cells (Figure 5M) and cortical astrocytes (Figure 5N) maintained the growth of neurites in almost all of the NEs analyzed in cell culture (98% for U87 cells and 100% for astrocytes) (Figure 5L) in contrast to the controls (Figure 5K). Through these experiments, we verified in vitro that neuron–glia interactions maintain DHA recycling and neurite outgrowth in cell cultures exposed to physiological concentrations of AA.

### 3.4. The Gradual Accumulation of DHA Impacts Redox Balance and Induces Protein Modifications in Adhered NEs

**Figure 6:**
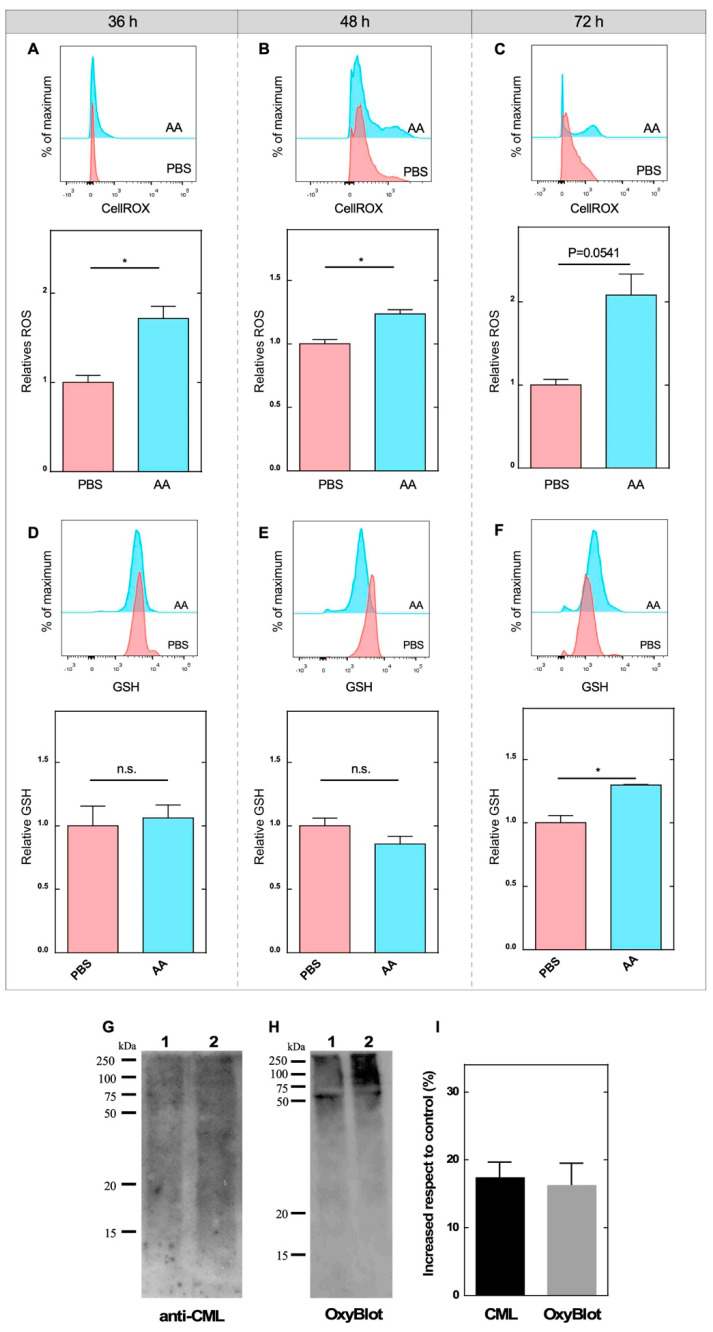
DHA accumulation impaired neurite outgrowth through redox imbalance. (**A**–**C**) Representative histogram of CellROX^®^ dye detection (APC channel) in control NEs (PBS, pink) and in NEs treated with 100 µM AA (light blue) for 36 (**A**), 48 (**B**) and 72 h (**C**), respectively. Fluorescence intensity quantification of CellROX^®^ in the control (PBS) and treated (AA) conditions is shown at the bottom. All data are representative of three separate experiments. Student’s *t*-test was used, * *p* < 0.05. (**D**–**F**) Flow cytometry analysis was performed using ThioBrightTM Green probe for intracellular GSH detection in control NEs (pink) and in NEs treated with AA (light blue) for 36 h (**D**), 48 h (**E**) and 72 h (**F**). The representative histogram of the detection of the probe through the FITC channel (GSH) is shown at the top. The quantification of fluorescence intensity in control samples (PBS) compared to treated samples (AA) at different times analyzed is shown in the lower part. Analysis was performed on three independent experiments; 20,000 events were recorded per condition. Student’s t-test was used, * *p* < 0.05; n.s., not significant. (**G**) Carboxymethyl-lysine (CML) immunoblot detection in control NE samples (lane 1) and NEs treated with AA (lane 2). All data are representative of three separate experiments. (**H**) Oxy-Blot analysis of protein carbonylation in extracts from control NEs (lane 1) and from those treated for 72 h with 100 µM AA (lane 2). All data are representative of three separate experiments. (**I**) Densitometry analysis of the CML and Oxy-Blot analyses is shown as a percentage of increase with respect to the control (PBS). All data are representative of three separate experiments.

To evaluate whether DHA generation can induce a redox imbalance that affects neurite growth in NEs, we used the CellROX probe to evaluate intracellular levels of reactive oxygen species (ROS) in NEs treated with AA for 48 h. We observed a significant increase in total ROS after 36 h of treatment compared to the control condition (Figure 6A); the same result was observed at 48 and 72 h (Figure 6B,C). Thereafter, we measured intracellular concentrations of GSH to evaluate the reducing capacity of these cells in response to the increase in ROS; no significant differences were observed at 36 and 48 h posttreatment (Figure 6D,E). However, at 72 h, a significant increase in GSH concentration was detected in the samples treated with AA (Figure 6E), confirming that NEs still have the capacity to induce the late biosynthesis of GSH.

Previous data indicate that DHA production and subsequent accumulation can induce protein modifications (increase in carboxymethyl-lysine, CML), such as the production of AGEs, which would reduce the outgrowth of neurites [27]. Thus, we analyzed CML by Western blotting, and we detected an increase in CML levels in cultured NEs treated for 72 h with AA compared with the controls (Figure 6G,I). In parallel, we analyzed other protein modifications (terminal and irreversible) that can occur when there is a high level of ROS, including protein carbonylation. Using Oxy-Blot, we detected a significant increase in the presence of carbonylated proteins in AA-treated samples, which were mainly located in high molecular weight proteins (Figure 6H,I). These observations suggested that the accumulation of DHA during prolonged treatment with AA induced a redox imbalance that favored an increase in ROS, inducing irreversible protein modifications, such as carbonylation, which could be a mechanism by which DHA affects neuritic growth. The increase in GSH concentrations at 72 h may be insufficient to prevent these modifications.

## 4. Discussion

In this work, we characterized an in vitro differentiation approach based on NSCs isolated from the embryonic cortex of an E17 rat, which formed NEs with a high number of βIII tubulin-positive cells when grown under adhesion conditions. Additionally, we found that NE cells with a commitment to neuronal differentiation also expressed the vitamin C transporter SVCT2 in the cytoplasm and neurites. This pattern of neuronal SVCT2 expression seems to reproduce what has been previously described in the cerebral cortex of postnatal rats, in which SVCT2 is expressed primarily in the pyramidal neurons of the deep layers of the cerebral cortex [16]. Different from previously described, our laboratory observed that the NEs isolated from the cerebellum and cultured in suspension expressed SVCT2 mainly in cells expressing nestin, an intermediate filament expressed in NSCs [12].

Interestingly, during the embryonic development of the cerebral cortex, RG cells positive for PCNA and 3-PGDH express SVCT2 in the apical region, which contacts the CSF, suggesting that vitamin C is an important molecule for stem/proliferative cells [22]. A similar situation was observed at the cerebellar level [12]. Thus, if NEs remain in suspension, they contain SVCT2/nestin-positive cells, which represents embryonic development and the SVCT2 expression pattern in the RG. However, if NEs are exposed to an adhesion substrate, they switch the differentiation process, with a large number of cells with a neuronal phenotype and with βIII tubulin-positive cells that express SVCT2, as previously described during the first days of the postnatal development of the cerebral cortex [16].

Although adhesion to a substrate is a stimulator of neuronal differentiation per se for NEs, we found that the presence of 100 µM AA during the first 48 h could stimulate the generation of a neuronal lineage with an increased neurite volume. Previously, our laboratory demonstrated that supplementation with physiological concentrations of AA increased the expression of neuronal markers such as βIII tubulin in NEs formed by P19 cells, which have NSC characteristics; the same was observed in NEs formed from the ventricular wall of adult rats [28]. Various lines of evidence corroborate that AA induces differentiation in precursor cells. Lee et al. [25] proposed that treatment with AA in cortical precursors not only increases neuronal differentiation but also increases the number of events and the amplitude of mEPSCs in cortical neurons, suggesting that AA stimulates neuronal maturation. Similarly, supplementation with AA during R1 cell differentiation induces an increase in the expression of neuronal differentiation genes, such as *Ntrk2* and *BDNF* [29]. Finally, the treatment of precursors isolated from the ventral midbrain with AA yields cells positive for TH, a classic marker of dopaminergic neurons, suggesting that ascorbate could regulate the differentiation of a specific neuron type [30]. The latter has been related to a cofactor role of AA in TET enzymes, including TET1 and JMJD3, which mediates the increased expression of dopamine genes, such as *Th* and *Nurr1* [31,32]. All these studies support our results that AA stimulates neuronal differentiation.

With respect to the different effects of RA and AA on adhered NE, AA surprisingly induced more neuritic processes in adhered NE, which contrasts with the known use of RA as a potent neuronal differentiator [23,33,34]. During embryonic development, RA is secreted by the meninges, inducing RG cell differentiation of cortical neurons [35]. In contrast, AA is highly concentrated during embryonic development in the cerebral cortex and CSF [36]; therefore, it is possible that both molecules exert simultaneous effects during embryonic development. In 2014, Wu et al. [37] compared the effect of RA and AA on the F9 cell line, which has characteristics of embryonic stem cells (ESCs), and observed that RA induced neuronal differentiation, while AA enhanced the undifferentiated phenotype by increasing the expression of the *Nanog* gene via JAK/STAT signaling. This evidence suggests that RA enhances the neuronal differentiation of embryonic stem cells, such as RG cells; in contrast, AA allows for the maintenance of pluripotency. The latter occurs during embryonic development; however, in more advanced stages when the cells have already acquired a neuronal phenotype, as in adhered NEs, AA enhances neuritic outgrowth more than RA.

Regarding the redox state of vitamin C in neurons, it has been found that neurons oxidize up to 80% of AA to DHA intracellularly within 1 h and efflux this molecule into the extracellular environment [18]. Therefore, the gradual accumulation of DHA in the cultures after 72 h of treatment was based on the low reducing capacity of the immature neurons that form the NE and that express SVCT2, in addition to the few mature astrocytes in the structure, which can reduce DHA to AA. Thus, adhered NEs were unable to establish a mechanism for vitamin C recycling. DHA supplementation in colon cancer cells decreased glycolysis and increased pentose pathway activity [10]. These same metabolic changes were described in cortical neurons exposed to DHA, in which a decrease in GSH levels and an increase in lactate uptake was also detected [18]. In addition, Scheffler et al. [27] described that DHA supplementation decreased the outgrowth of neurites in PC12 cells differentiated with NGF; however, the impact of vitamin C recycling was not evaluated. Therefore, for the first time, we reported how sustained DHA accumulation can alter neuronal morphology and neurite growth.

DHA supplementation in cortical neurons previously exposed to H_2_O_2_ induced neuronal death, which was reversed in the presence of cortical astrocytes that capture DHA [19,38]. Therefore, to date, the function of vitamin C recycling at the neuronal level has only been described in the context of the maintenance of metabolism and neuronal viability. In this work, we propose for the first time that coupling occurs between immature neurons and cells characterized by their high potential to capture DHA, namely, HL60 cells [8], U87 cells or cortical astrocytes [19]. This coupling is necessary for the maintenance of neuritic structures in the presence of AA. Although our laboratory showed that the expression of SVCT2 and the uptake of AA can enhance the generation of lamellipodia and filopodia in neuroblastoma cells [17], no previous evidence attributes a regulatory function of neuronal morphology for DHA. In saline buffer, DHA has a half-life of up to 50 min [39]; however, at high concentrations or in the context of oxidative stress, it irreversibly oxidizes to 2,3-diketogulonic acid (2,3-DKG), which can be broken down into byproducts that derive from the fragmentation of the 6-carbon main chain [40]. These carbonyl products are highly reactive, and under conditions of oxidative stress, they can carry out the Maillard reaction, generating terminal glycation products, such as N^ε^- [carboxymethyl]-lysine (CML) and pentosidine in the lens [41]. Recently, it has been described that both AA and DHA can increase CML levels during neuronal differentiation [27]; it has also been suggested that DHA could bind GSH [42], mediate the S-thionylation of glutaredoxin and homocysteine [43] and induce S-glutathionylation of GAPDH [10]. Thus, DHA has the ability to induce protein modifications that alter the function of antioxidants and peptides that contain amino acids with a thiol group, such as cysteine. In our model, DHA accumulation induced an oxidative stress environment, which was indicated by the increase in ROS. Redox imbalance can affect the dynamics of the cytoskeleton, which can regulate the growth or shortening of processes or the loss of neuritic processes depending on the dynamics of actin and tubulin. The actin monomer contains 5 cysteine residues and 16 methionine residues, while α and β tubulin have 12 and 8 cysteine residues, respectively, which are susceptible to oxidation [44]. In particular, glutathionylation of Cys 374 in G-actin induces a structural change that slows the addition of the monomer to the growing filament [45]. Therefore, our results suggest that DHA accumulation triggers terminal protein modifications, such as CML generation and carbonylation, which could affect cytoskeletal proteins, inhibiting the maintenance of neurites. This effect is hypothesized to be reversed by the establishment of vitamin C recycling between immature neurons and young astrocytes, a mechanism that would allow the regulation of neuronal morphology in the postnatal cerebral cortex. In this work, we used NEs formed from NSCs that, when grown under adhesion conditions, differentiate and induce immature neurons that express SVCT2, a model of the early postnatal development of the cerebral cortex.

## 5. Conclusions

We demonstrated that treatment with AA enhanced neuronal differentiation; however, the gradual accumulation of DHA induced neurite loss mediated by an oxidative environment that induced irreversible protein modifications. The presence of DHA recycling, established by cortical astrocytes, allowed for the maintenance of neurites. Thus, this is the first report describing vitamin C recycling as a mechanism that regulates neuronal morphology during neuronal differentiation and maturation.
